# Rice immune sensor XA21 differentially enhances plant growth and survival under distinct levels of drought

**DOI:** 10.1038/s41598-020-73128-7

**Published:** 2020-10-09

**Authors:** Shamsunnaher, Xiuhua Chen, Xiaoxuan Zhang, Xiao-Xia Wu, Xiaoen Huang, Wen-Yuan Song

**Affiliations:** 1 Department of Plant Pathology, University of Florida, Gainesville, FL 32611 USA; 2 Bangladesh Rice Research Institute, Gazipur, 1701 Bangladesh; 3 Benson Hill Biosystems, St. Louis, MO 63132 USA; 4 College of Horticulture, Northeast Agriculture University, Harbin, 150030 China; 5 College of Bioscience and Biotechnology Yangzhou University, Yangzhou, 225009 China; 6 Citrus Research and Education Center, University of Florida, Lake Alfred, FL USA

**Keywords:** Genetics, Immunology, Molecular biology, Plant sciences

## Abstract

Drought is a complex stress that limits plant growth and crop production worldwide. The mechanisms by which plants coordinately respond to distinct levels of water deficits (e.g., mild, moderate or severe drought) remain elusive. Here we demonstrate that the rice immune sensor XA21 promotes survival of rice seedlings during dehydration stress. XA21 expression increases deposition of lignin and cellulose in the xylem vessels and their surrounding cells. Inhibition of aquaporin water channels by mercuric chloride eliminates XA21-mediated dehydration survival, suggesting that XA21 enables plant survival during drought, probably by protecting xylem functionality. In contrast to prevailing observations of stress tolerance genes, XA21 is also capable of enhancing rice growth during moderate drought. Thus, XA21 acts as a mediator for stress protection and plant growth under water-limiting conditions.

## Introduction

Terrestrial plants have evolved various strategies to adapt to the amount of water available in their environment. For example, vascular plants can grow to larger sizes due in part to their xylem, which efficiently transports water from roots by transpiration. When drought causes xylem wall collapse or embolism (gas bubble), water movement is impeded, which can cause tissue damage and even plant death^1–3^. One way to protect water transport capacity under stress is the production of thicker secondary cell walls, composed mainly of hydrophilic cellulose and hydrophobic lignin, that provide rigidity and mechanical support to the xylem. It has also been hypothesized that higher contents of lignin increase plant resistance to embolism^4^. Following its axial movement through the xylem, water is delivered from the xylem to the mesophyll cells through radial flow, which may require the activity of aquaporin (AQP) water channels^5^. During drought stress, AQPs are thought to play an important role in refilling embolized xylem conduits by using water from the surrounding parenchyma cells^5,6^.

Many plants rapidly reduce their growth rates under mild to moderate drought^7^. Both whole-genome transcription profiling and genetic studies from Arabidopsis have suggested that mechanisms regulating survival under severe drought stress differ from those controlling growth during mild to moderate water deficits^8–11^. However, little is known about either the higher-order controls of complex drought responses in plants or the genes regulating both survival and growth under water-deficit conditions. Tight control of these responses are crucial, as constitutive activation of drought protective mechanisms can result in a growth penalty^12^.

Rice is a staple food for more than 50% of the human population and an important model of the monocotyledons in the family Poaceae, which includes other major food and bioenergy crops (e.g., maize, wheat and sorghum). Rice crop residues represent 50% of the agronomic biomass produced worldwide^13^. However, rice is sensitive to various pathogen infections as well as water-deficit stress. The Gram-negative bacterium *Xanthomonas oryzae* pv. *oryzae* (*Xoo*) proliferates in the xylem vessels, causing bacterial leaf blight disease of rice^14^. Typical symptoms include leaf rolling and wilting reminiscent of those seen in plants stressed by water deficit^15^. The rice receptor kinase XA21 confers immunity to *Xoo* in adult plants by perceiving a pathogen- or microbe-associated molecular pattern^16,17^. We reported previously that XA21 resistance can be fully activated in 2-week-old seedlings by a low temperature (23–27 °C) treatment, but abolished by higher temperatures (31 °C or above)^18^. In this study, we investigate the capacity of the immune receptor XA21 to function as a drought mediator.

## Results

### XA21 enhances plant survival after dehydration stress

**Figure 1:**
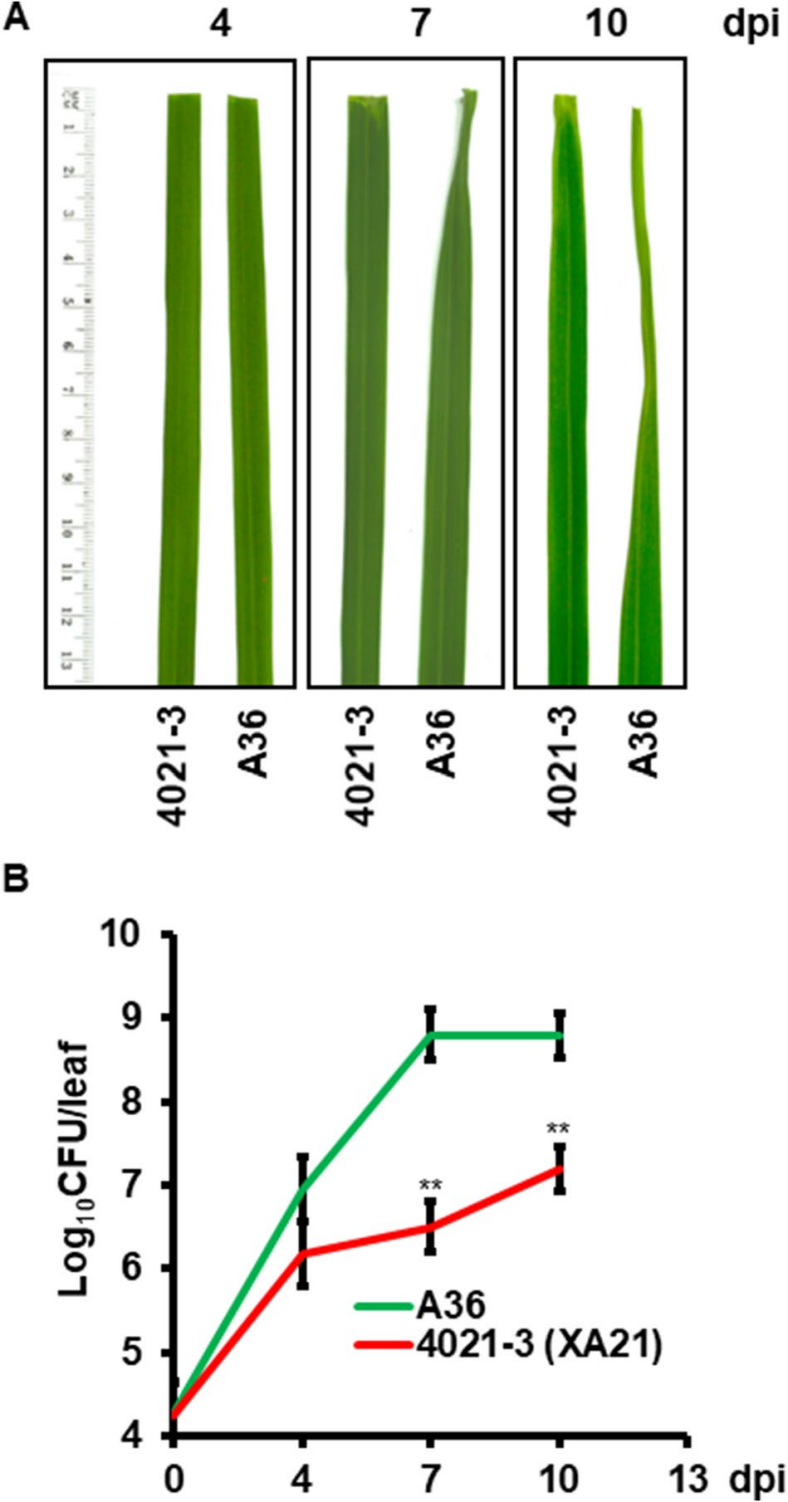
XA21 plants display milder drought-related symptoms than the control after *Xoo* infection. (**A**) Phenotypes of representative 6-week-old A36 (empty-vector control) and 4021-3 (expressing Myc-XA21) leaves after inoculation with the incompatible *Xoo* strain PXO99^A^; dpi, day post inoculation. (**B**) Growth of PXO99^A^ in A36 and 4021-3 leaves. CFU, colony-forming units. Error bars are SD (n = 6); **, *P* < 0.01.

When the previously characterized transgenic rice line 4021-3, expressing XA21 fused to an N-terminal c-Myc tag (Myc-XA21)^19^, was inoculated with the incompatible *Xoo* strain PXO99^A^, the bacterium propagated to a significant level; but only caused short disease lesions and weak water stress symptoms (e.g., leaf rolling) compared to empty vector-carrying line A36 (Fig. 1). We reasoned that XA21 might aid rice in adapting to a water-deficient environment in addition to its ability to limit *Xoo* proliferation.

**Figure 2:**
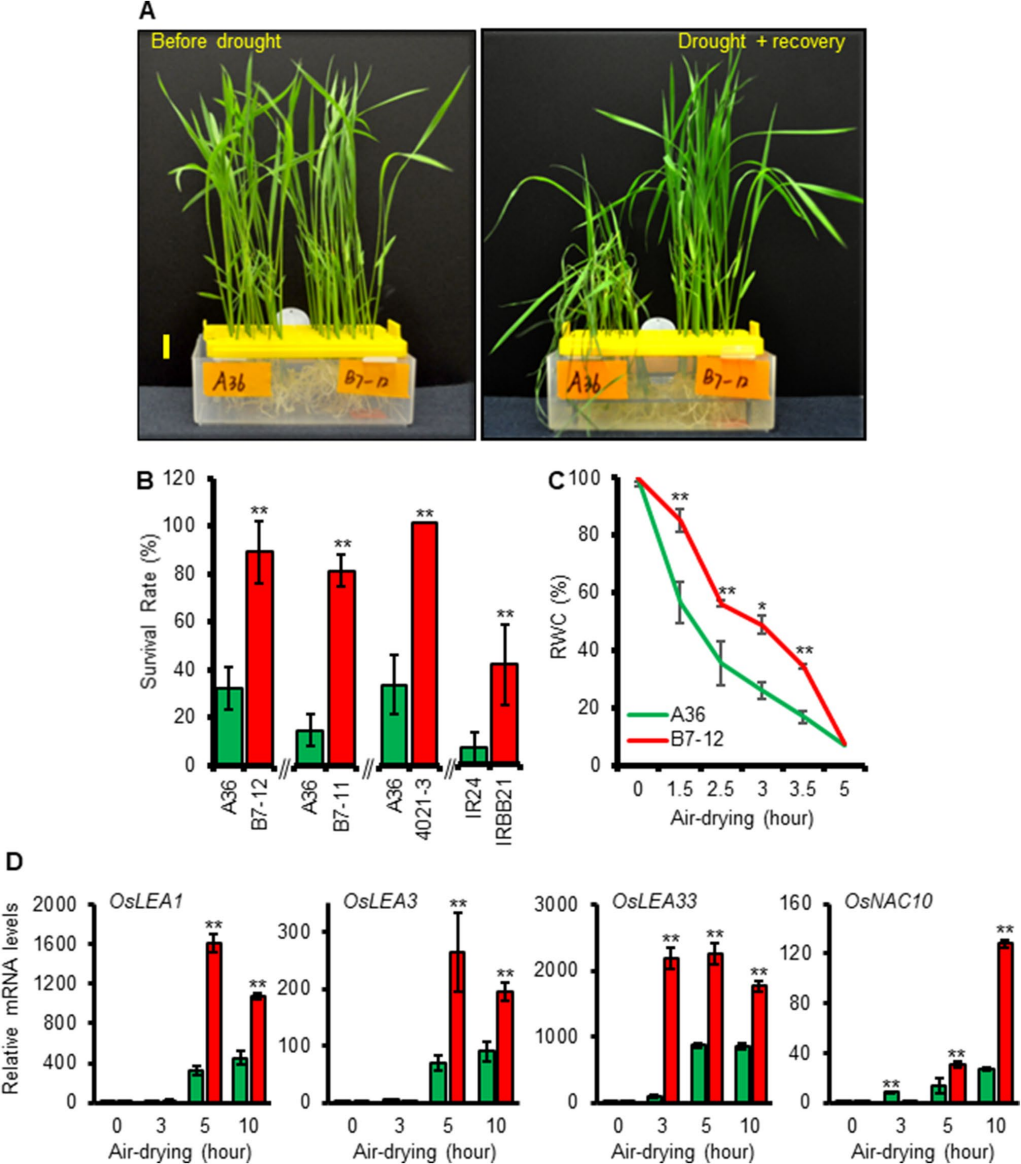
XA21 is required for enhanced survival of rice seedlings under dehydration stress. (**A**) Phenotypes of representative 2-week-old A36 (empty-vector control) and B7-12 (expressing 3xFLAG-XA21-Myc) plants (n = 24 per line) prior to and after dehydration stress. (**B**) Survival rates of dehydration-treated XA21 (B7-12, B7-11, 4021-3 and IRBB21) and control (A36, IR24) lines (n = 24 per line). (**C**) Relative water content (RWC) of A36 and B7-12 seedlings over 4 h of dehydration. Scale bars (yellow) in A = 2 cm. Error bars in (**B**) and (**C**) are SD (n = 3). *, *P* < 0.05; **, *P* < 0.01. (**D**) XA21 potentiates expression of drought responsive genes in response to dehydration stress. Transcript data of four drought responsive genes, as measured by q-PCR, represent the mean ± SD of three biological replicates. **, *P* < 0.01.

We first tested the ability of XA21 to promote survival during dehydration. Newly generated homozygous XA21 lines (B7-12 and B7-11), expressing 3xFLAG-XA21-Myc under the control of its native promoter, conferred resistance to *Xoo* PXO99^A^, whereas the empty-vector control line A36 was susceptible (Supplemental Fig. 1). Air-drying of 2-week-old seedlings for 3.5 h (h) at 23 °C caused > 54% mortality in A36, but < 25% death in lines B7-12, B7-11 and 4021-3 (Fig. 2A,B). The introgression line IRBB21 (the original source used to isolate the *Xa21* gene)^20^ also exhibited better performance under dehydration relative to the near-isogenic recurrent parent *O*. *sativa* ssp. *indica* cv. IR24 (Fig. 2B). Under well-watered conditions, the relative leaf water content (RWC) of the two genotypes were similar. The RWC was sharply reduced by dehydration in both the B7-12 and A36 lines, but with a statistically larger decrease in the control seedlings (Fig. 2C). These results indicated that XA21 is indeed capable of improving the survival of rice during dehydration stress, and these transgenic lines provide a basis to further characterize this function of XA21.

*LEAs* encode hydrophilic proteins that potentially function in cellular protection during water deficit^21^, whereas *OsNAC10* codes for a transcription factor of the NAM ATAF CUC2 (NAC) family^22^. Overexpression of *OsLEA3* or *OsNAC10* enhances drought tolerance in rice^22,23^. Members of the *LEA* family and *OsNAC10* are well-known to be induced by drought stress^22,24^. We selected *OsLEA1*, *OsLEA3*, *OsLEA33* and *OsNAC10* as drought responsive markers to exame their expression patterns between B7-12 and A26 during dehydration stress using reverse transcription-quantitative polymerase chain reaction (q-PCR). As shown in Fig. 2D, all four genes were induced by dehydration treatment and expressed higher levels in B7-12 than A36. Together, these data indicated that, in response to dehydration stress, XA21 triggers heightened expression of a battery of drought-responsive genes.

### XA21 plants accumulate higher levels of cellulose and lignin in leaf vascular tissues

**Figure 3:**
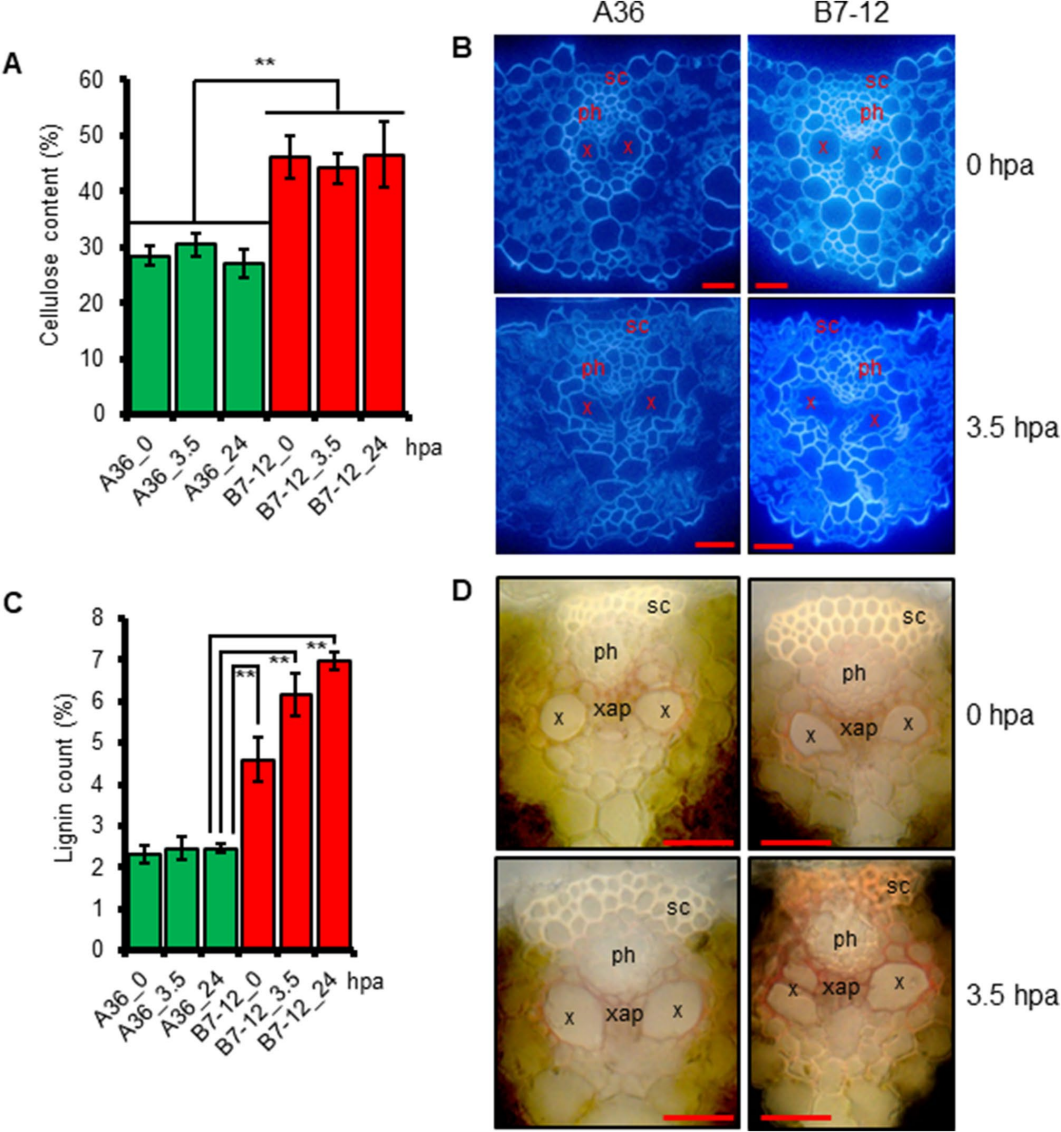
XA21 is required for increased accumulation of cellulose and lignin in rice leaf tissues. (**A**) Leaf cellulose content of indicated genotypes at 0, 3.5 or 24 h post air-drying (hpa). (**B**) Cross sections of leaf blades of indicated lines stained with calcofluor white (binding with cellulose) at indicated time points. X, xylem; ph, phloem; sc, sclerenchyma cells. (**C**) Leaf lignin content at indicated time points. (**D**) Cross sections of leaf blades of indicated lines stained with phloroglucinol (staining lignin polymers in red). X, xylem; ph, phloem; sc, sclerenchyma cells; xap, xylem associated parenchyma cells. Scale bars in (**B**) and (**D**) = 20 μm. Error bars in (**A**) and (**C**) are SD (n = 4). (**) *P* < 0.01.

Xylem structure and function are important for plant response to water stress. In 2-week-old seedlings, biochemical quantification showed that B7-12 leaves accumulated higher levels of cellulose compared to A36 (Fig. 3A, at the 0 time point) and that these levels were maintained during 24 h of drought stress. The increased cellulose deposition occurred mainly in cell walls of the vascular tissues (Fig. 3B).

We also detected higher lignin content before dehydration in B7-12 than in the control (A36) (Fig. 3C). Different from cellulose accumulation, lignin levels in XA21 leaves were further increased after stress. Lignin accumulated mainly in sclerenchyma and the parenchyma cells adjacent to the xylem vessels of B7-12 seedlings (Fig. 3D). Together, these data suggested that XA21 function may have a role in strengthening xylem structure.

### Expression of XA21 aids in the restoration of xylem function after dehydration stress

**Figure 4:**
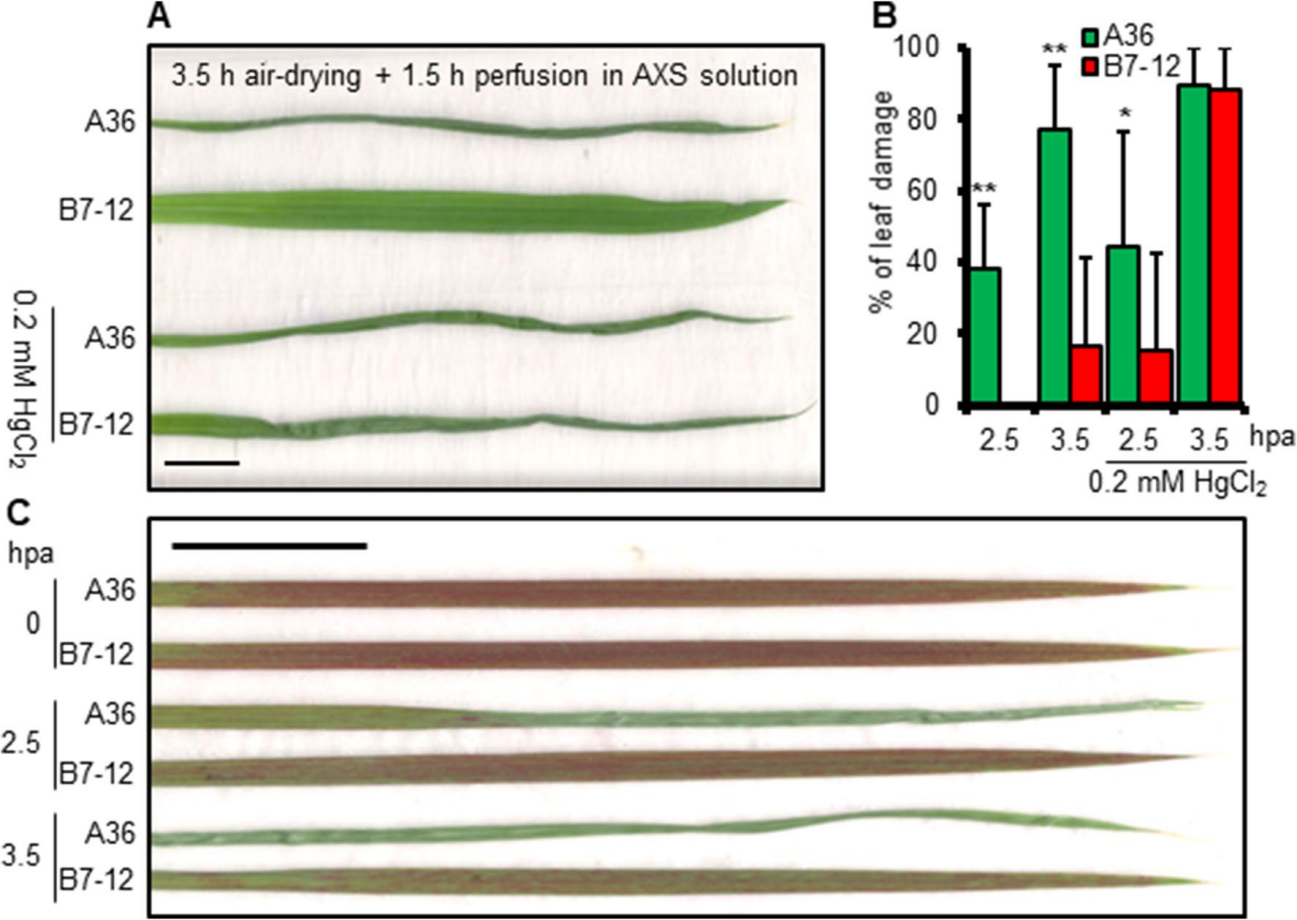
XA21 is responsible for improved xylem refilling after dehydration. (**A**) Effect of mercuric chloride (HgCl_2_) on dehydration recovery of A36 and B7-12 leaf blades. (**B**) Percentage of leaf damage after dehydration recovery with or without HgCl_2_. Hpa, hours post air-drying. Error bars are SD (n = 10). (*) *P* < 0.05 (**) *P* < 0.01. (**C**) Typical images of rice leaves dehydrated for the indicated time periods followed by perfusion with safranin solution for 1.5 h by transpiration. The experiments were repeated more than three times with similar results. Scale bars in (**A**) and (**C**) = 1 cm.

Refilling of embolized xylem vessels during drought requires water supply from the surrounding cells, and AQPs are considered to be the key channels of this water transport^5^. To demonstrate that AQP function is essential for XA21-mediated dehydration survival, we treated rice leaves with mercuric chloride, a widely used inhibitor of AQPs, using a detached leaf assay published previously^25^. Leaves were excised from seedlings pre-hydrated for 3.5 h and then their cut-ends were immersed into artificial xylem sap (AXS). Those from B7-12, but not A36, uncurled in the absence of mercuric chloride (Fig. 4A). The recovery of the B7-12 leaves occurred within 5–10 min, suggesting that the water transport ability in the XA21 line may not be disrupted by dehydration-induced embolisms, thereby allowing quick delivery of water to the mesophyll cells in the recovery phase. By contrast, the feeding of mercuric chloride into the transpiration stream from the cut-ends largely blocked the recovery of the pre-dehydrated B7-12 leaves (Fig. 4A,B). Increased recovery was observed when the two genotypes were stressed for a shorter time period (i.e., 2.5 h), although B7-12 leaves did perform better than A36. Thus, AQP function during dehydration is likely required for XA21 leaves to survive a longer time period of severe water stress.

Safranin uptake was used to assess the role of XA21 in xylem refilling after dehydration treatments. The dye moves in the transpiration stream to stain the xylem elements, and serves as a tool to trace water transport in the conduits^25^. Safranin was readily visible in whole veins of the leaves excised from unstressed A36 and B7-12 seedlings 1.5 h after dye perfusion (Fig. 4C and Supplemental Fig. 2). However, A36 leaves dehydrated for 2.5 or 3.5 h showed very limited dye staining, indicative of irreversible impairment of xylem function induced by dehydration stress in the control line. Accordingly, large areas on the distal half of the stressed leaves were unable to recover from the stress. By contrast, safranin stained most of each B7-12 leaf subjected to the same duration of dehydration stress, despite a reduction in the density of stained vessels compared with leaves from unstressed seedlings. These findings, in combination with the quick recovery of B7-12 leaves described above, suggested that XA21 contributes to the maintenance of xylem function during dehydration, consequently facilitating the restoration of water transport in the recovery phase.

### XA21 enhances rice growth under moderate water deficit

**Figure 5:**
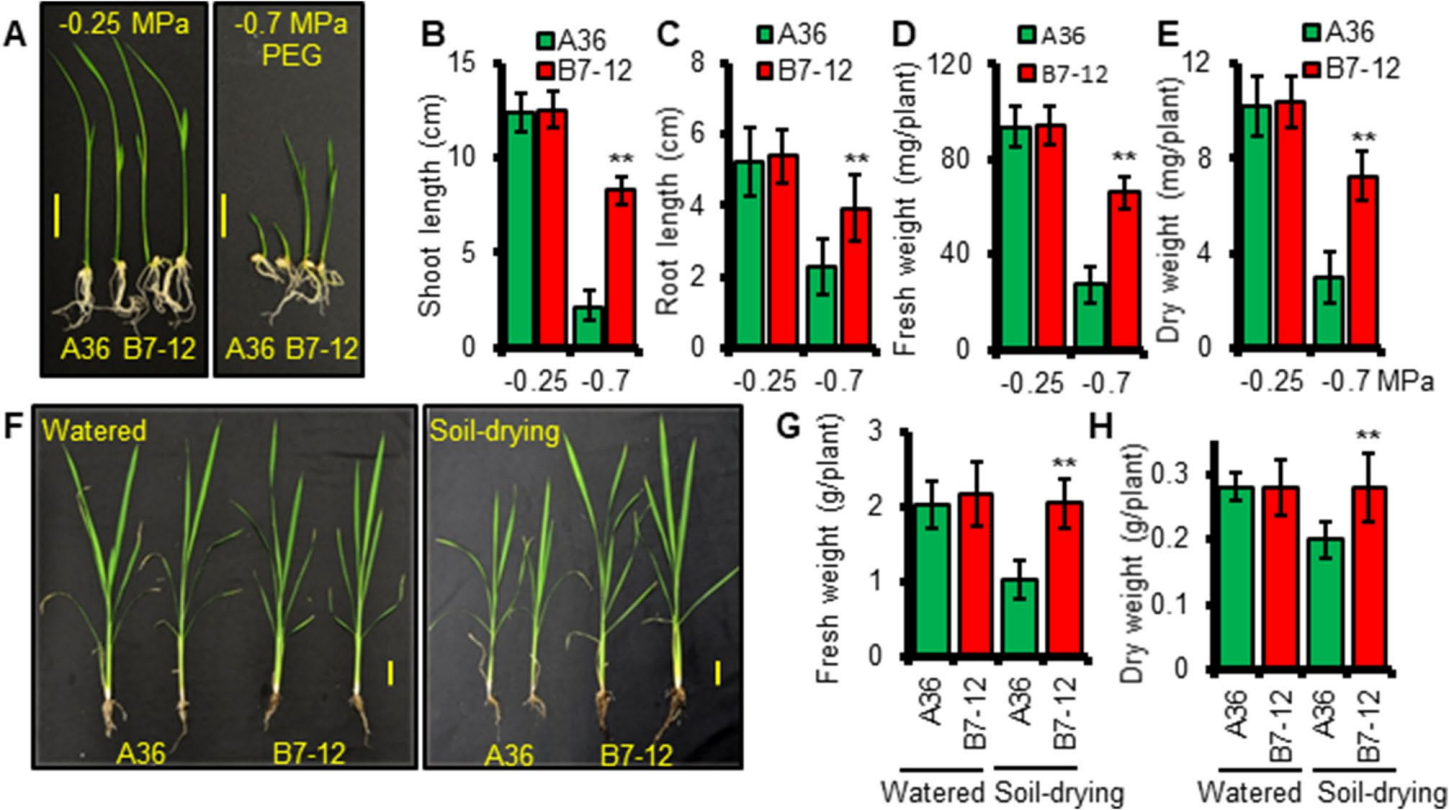
XA21 is required for enhanced growth under moderate water deficit. (**A**) Phenotypes of representative A36 and B7-12 seedlings grown on control (ψ_w_ =  − 0.25 MPa) or PEG-infused low water potential (ψ_w_ =  − 0.7 MPa) medium. (**B**,**C**) Shoot and root lengths of seedlings grown on low-ψ_w_ medium. (**D**,**E**) Fresh and dry weights of seedlings grown on low-ψ_w_ medium. (**F**) Phenotypes of representative plants grown in well-watered soil or partially dry soil for one month. (**G**,**H**) Fresh and dry weights of plants treated with moderate drought. Scale bar in (**A**) = 2 cm. Scale bar in (**F**) = 4 cm. Error bars in (**B**–**E**) (n = 10) and in (**G–H**) (n = 3) are SD. (**) *P* < 0.01.

We next tested whether XA21 is capable of influencing plant growth under mild to moderate water deficits since *Xoo*-infected rice leaves likely face dynamic water availability. As expected, the growth of A36 seedlings was reduced dramatically when transferred from half strength MS medium to a low-water potential (low-ψ_w_), PEG-infused medium (− 0.7 MPa) (Fig. 5A). XA21 seedlings (B7-12, B7-11, 4021-3, IRBB21) displayed significantly better growth, as measured by the fresh and dry weights of both shoots and roots, under the same low-ψ_w_ conditions (Fig. 5A–E and Supplemental Fig. 3). Soil drying experiments showed that under moderate levels of drought stress, imposed by keeping the soil matric potential (SMP) between − 300 to − 900 kPa, B7-12 plants showed a growth advantage, as judged by fresh and dry weight, over the control line one month after stress treatments (Fig. 5F–H and Supplemental Fig. 4). B7-12 had longer leaves than A36, starting with the 4th leaf (Supplemental Fig. 4B). No significant difference in growth was observed when both B7-12 and A36 were grown under well-watered conditions (Supplemental Fig. 4C).

### XA21 plants display drought resistance under greenhouse conditions

**Figure 6:**
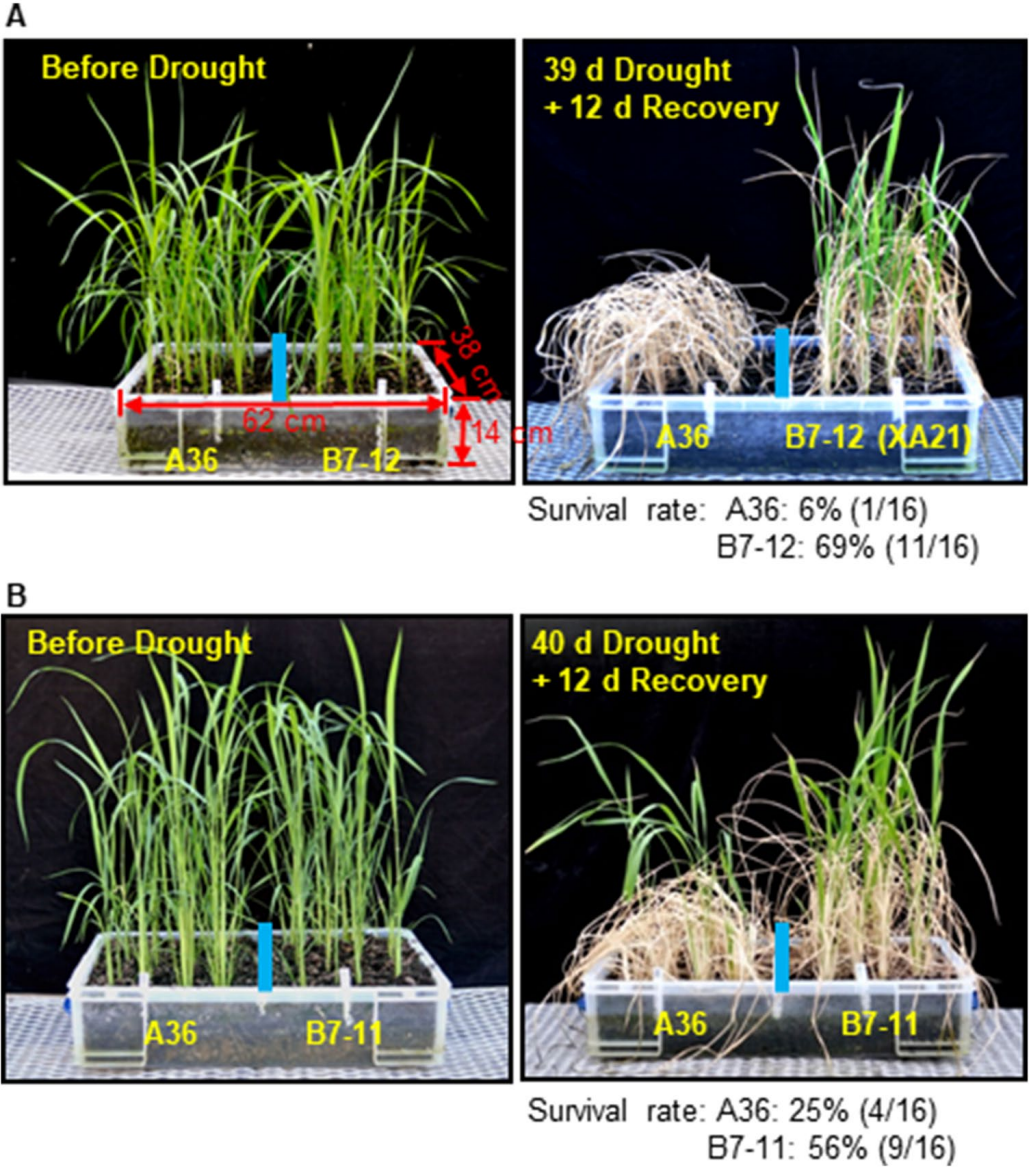
XA21 plants display enhanced drought survival under greenhouse conditions. Phenotypes of 1-month-old XA21 plants [B7-12 (**A**) or B7-11 (**B**)] and control (A36) (n = 16 each line) before and after drought stress (withheld water). The survival rates for each line are indicated.

We also performed drought treatments by withholding water using one-month-old plants grown in a greenhouse. Leaf rolling is an early, visible symptom of drought stress in rice and other grasses^26,27^. A36, not B7-12, leaves started rolling 22 days after cessation of watering (Supplemental Fig. 5). Between days 28–30 of the stress period, many leaves on A36 plants turned gray and later brown, indicating that massive tissue death occurred in the control line. In contrast, B7-12 leaves remained green although they also rolled during this stage. When the stress continued for an additional nine days, leaf death extended to all A36 plants, but the rolled B7-12 leaves started turning gray and brown. As expected, significantly more B7-12 plants were recovered than A36 plants after rewatering (Fig. 6A). Similar results were obtained for the XA21 lines B7-11 and 4021-3 (Fig. 6B, Supplemental Fig. 6A). Of note, the drought resistance appeared to be influenced by the seasons (likely due to temperature changes) and by developmental stages with greater degree in winter and at the older stages. We also noticed that vegetative growth of 4021-3 plants with a relative higher level of XA21 protein expressed by its native promoter^19,28^ appeared to be slightly retarded in winter but not in other seasons (Supplemental Fig. 6B).

## Discussion

Plants lack an adaptive immune system like those used by mammals to eliminate infectious microbes. This results in a significant titer of pathogens remaining inside the host for an extended period of time even a lifespan. Because cumulative growth of *Xoo* in the xylem vessels would cause progressively increasing water deficits in rice leaves, we hypothesize that the immune sensor XA21 not only restricts bacterial over-accumulation, but also serves as a mediator controlling distinct levels of drought response. To support this hypothesis, we demonstrated that XA21 is capable of improving rice performance under both moderate drought and severe dehydration conditions. It has been shown that another immune receptor, chitin elicitor receptor kinase 1 (CREK1), can enhance Arabidopsis tolerance to salt stress, and this novel function has been proposed to be mediated by detecting the elevated Na^+^ ions induced by fungal attack^29^. Thus, plant immune receptors might play a broader role in perceiving complex environmental changes during pathogen infections.

Unlike many drought survival genes, XA21 assists in rice growth under moderate water stress. In the infected leaves, *Xoo* populations are inversely proportional to the distances from the inoculation site^30^. Therefore, cells at different locations of the leaves would experience distinct levels of water stress. Mild to moderate drought often causes a rapid delay of plant growth, although this acute growth inhibition is unlikely due to carbon limitation^7,10,31^. To reduce the disturbance of stress, genes enabling plant growth during sporadic occurrence of moderate water stress can be favorable^7^. We hypothesize that the drought function of XA21 may have a role in maintaining rice growth under the water stress conditions caused by *Xoo* infection.

A mechanism underlying XA21-mediated dehydration survival is maintenance of xylem function under water stress. This is likely achieved by tailoring cell wall composition and increasing transcellular water movement to refill embolized xylem vessels. We observed increased cellulose and lignin contents in the leaves of 2-week-old XA21 seedlings prior to dehydration stress and further enhanced accumulation of lignin under stress. The difference in the accumulation of cellulose and lignin in response to dehydration stress may reflect the distinct regulation of the biosynthesis of these two cell wall components by XA21 signaling at the seedling stage. Deposition of lignin and cellulose have been reported during incompatible interactions of *Xoo* with rice lines containing the resistance genes *Xa4*, *Xa10* and *Xa27*^14,32,33^. Furthermore, the presence of *Xa4*, also encoding a receptor-like kinase protein similar to XA21, positively influences cellulose, likely not lignin, deposition even in the absence of *Xoo*^34^. At the heading stage of rice growth, XA21 can affect the expression of numerous genes prior to infections, which has been suggested as a way to prime the immune system to efficiently fight *Xoo*^35^. Therefore, the observed accumulation of cellulose and lignin is likely the result of activation of XA21 signaling during development and as well as in response to stress.

We observed drought resistance from multiple XA21 lines in soil drying experiments under greenhouse conditions. The resistance was apparently affected by high temperature, which is in line with our previous observation that a 4 °C temperature shift from 27 to 31 °C abolishes XA21-mediated immunity at the juvenile stage^18^, but differs from the fact that high temperature treatments do not seemly affect XA21-mediated resistance against *Xoo* at the adult stage. This may reflect a difference between XA21-mediated immune and drought signaling in adult plants.

In light of our findings, we propose that, in response to varying degrees of water shortage, XA21 is capable of reprogramming distinct cellular processes for adjustment of the drought response. This system, which may have evolved from the pressures of pathogens such as *Xoo*, provides a novel approach for understanding the adaptations of vascular plants to variable water limitations. The knowledge revealed here will also aid the genetic engineering of drought resistance in crops.

## Materials and methods

### Plant materials

Rice (*Oryza sativa* L.) subspecies *japonica* cv. TaiPei309 (TP309), *O*. *sativa* ssp. *indica* IR24, and their derivatives were used in this study. Seeds with similar vigor were surface-sterilized with bleach and germinated on half-strength Murashige-Skoog (MS) medium supplemented with 30 g/L sucrose and 50 µg/ml hygromycin (for transgenic *japonica* lines only) for 9 days in a growth room with a 16 h photoperiod, a light intensity of 160–180 µm photons m^−2^ s^−1^ and at 23–25 °C. Germinated seedlings were either grown in a greenhouse or cultured in water until stress treatments or *Xoo* inoculation as described below.

### Rice inoculation and disease evaluation

Two-week-old seedlings (grown in medium) or 6-week-old plants (grown in soil) were inoculated with the *Xoo* strain PXO99^A^ as described^19^. The seedlings were cultured for 12 days after inoculation for disease development in an incubator as above but at 27 °C. Inoculated adult plants were maintained in a growth room as above between 26 and 30 °C. Disease lesion and bacterial population were determined as described^19^.

### Plasmid construction

The 3xFLAG-XA21-Myc construct was made using an 9.9-kb genomic fragment, containing the *c*-*Myc*-tagged *Xa21* coding region, intron (not shown) and the native 5′ and 3′ regulatory sequences, previously used for rice transformation (Supplemental Fig. 1)^19^. To delete the extra 3′ sequence from the 9.9 kb *Xa21*-containing fragment, a *Kpn*I-*Spe*I fragment with *Myc*-*Xa21* was mobilized from the plasmid pBEK822-Bm into the vector pKBluescript to generate pKBXA21KS-M. An additional 1.8 kb 3′ sequence, PCR amplified from the 9.9 kb *Xa21* fragment with primers XA21-Tail-F/-R (Supplementary Table 1), was added to the 3′ end of the *Kpn*I-*Spe*I fragment of pBXA21KS-M using the *Spe*I site. The resultant construct, pKB-Myc-XA21-S, contains a c-Myc tag in the N-terminal region (domain B) of XA21. To introduce a c-Myc tag to the C-terminus of XA21, the *EcoR*I fragment of pKB-Myc-XA21-S was replaced by one with the tag fused to the C-terminus of XA21. The N-terminal c-Myc tag in the construct was replaced with 3xFLAG using the *Dra*III site. The 8.7-kb *Kpn*I fragment containing *Myc-Xa21-3xFLAG* was verified by DNA sequencing and subcloned into the binary vector pCAMBIA1300. *Agrobacterium*-mediated transformation was performed using rice cultivar TP309 as described^19^.

### Immunodetection

Protein extraction and protein blot analysis were performed as described^19^.

### Stress treatments

For dehydration assays, 11-day-old (for *indica* lines IRBB21 and IR24) or 2-week-old (for all *japonica* lines) seedlings were air-dried in a growth chamber (23 °C) for the indicated time followed by a recovery in liquid half-strength MS medium for three days. Survivors were defined as individuals that had at least one uncurled true leaf after recovery.

We determined the RWC of dehydration-stressed leaves using the equation: RWC = (FW − DW)/(TW − DW), where FW is the fresh weight of the leaf discs collected. Turgid weight (TW) was measured after floating the leaf discs on water for 24 h at room temperature in dark. Dry weight (DW) was determined by weighing the leaves after drying at 65 °C for 3 days.

For HgCl_2_ treatments of detached rice leaves, 2-week-old seedlings of B7-12 and A36 lines were air-dried for the indicated times at 23 °C. The 2nd leaves of the stressed seedlings were excised under water, and the cut side was immersed into artificial xylem sap (AXS: 1 mM KH_2_PO_4_, 1 mM K_2_HPO_4_, 1 mM CaCl_2_, 0.1 mM MgSO_4_, 3 mM KNO_3_ and 0.1 mM, MnSO_4_ buffered to pH 5.8 with 1 M HCl or KOH) or AXS containing 200 mM HgCl_2_. AXS uptake was allowed for 1.5 h under light-emitting diode (LED) lights (1200 µm photons m^−2^ s^−1^) at 23–25 °C in the growth room. Leaf damage was defined as the length of shrunken plant tissues from tips.

Dye uptake experiments using detached rice leaves were as the HgCl_2_ treatments except that 0.1% (w/v) safranin was used in stead of HgCl_2_.

PEG stress assays were carried out as described^36^, with some modifications. Briefly, rice seeds with similar vigor were germinated on half-strength MS medium (containing no sucrose) supplemented with 50 µg/ml hygromycin for 3 days. Germinated seedlings were then transferred onto freshly prepared PEG-infused agar plates (containing no sucrose nor hygromycin, − 0.7 MPa) or control medium (− 0.25 MPa) for additional 5 days. Growth parameters were then scored.

To assess the growth performance of plants under mild to moderate water-deficit stress in soil, 2-week-old seedlings (germinated on ½ MS medium) were planted in containers [21.5 × 15.5 × 9.5 cm (L × W × H), three plants each genotype in one container] with pre-wetted soil (Supplemental Fig. 4A). Plants were maintained in the growth room as above. An MPS-6 water potential sensor (Decagon Devices) was embedded into soil to monitor the soil matric potential (SMP) every 60 min for the entire period of plant growth. Re-watering was carried out periodically to keep the SMP between − 300 to − 900 kPa. Growth parameters were recoded one month after transplanting.

Germinated seedlings were transferred into soil and grown in shared soil-holding trays prepared with evenly distributed holes on the bottom for absorbing water. The trays were maintained in large tanks filled with water in a greenhouse under nature light conditions in Gainesville, Florida. For drought treatments, the plant trays were transferred to a bench and kept under natural light conditions without watering for approximately 20–40 days depending on the season. To recover drought-stressed plants, the trays were returned to water tanks for 12 days before survivors were scored.

### Total RNA extraction and q-PCR analysis

Two-week-old seedlings of A36 and B7-12 were subjected to dehydration stress treatment. Total RNA was extracted from the leaf tissues harvested from the stressed seedlings using TRIzol (Ambion) according to the manufacturer’s instructions. The isolated RNA samples were then treated with RNase-free DNase (Qiagen) to eliminate genomic DNA contamination followed by further purification using the RNeasy MinElute Cleanup Kit (Qiagen). Single stranded cDNA was synthesized with 1 µg of total RNA using a RT2 First Strand Kit (Qiagen). Q-PCR was performed under the following conditions: 95 °C, 2 min; (95 °C, 5 s; 60 °C, 5 s) × 40 cycles, 72 °C, 5 min. using the CFX 96 Real-Time PCR Detection System (Biorad) according to the manufacturer’s instructions. Results were normalized to the expression of the rice reference gene Os06g11170^37^. Primers used for q-PCR are listed in Supplementary Table 1.

### Quantification of lignin

Lignin content of rice leaves was quantified according to the thioglycolic acid method described previously^38^. In brief, the 2nd leaves were harvested from 2-week-old seedlings prior to and after dehydration stress. The prepared cell wall samples were dried, weighted and mixed with a reaction mixture containing 0.1 ml of thioglycolic acid (Sigma) and 1 ml of 3 N HCl. The samples were then incubated at 80 °C for 3 h. After centrifugation, the pellet was collected, washed once with distilled water and dissolved in 1 ml of 1 N NaOH. Following acidification with 0.2 ml of concentrated HCl for 4 h at 4 °C, the samples were dissolved in 1 ml of 1 N NaOH. Diluted samples were subjected to spectrophotometric measurements.

### Quantification of cellulose

Cellulose content of rice leaves was measured as described^39^. Briefly, the 2nd leaves were harvested from 2-week-old seedlings prior to and after dehydration stress for preparation of the alcohol insoluble residue, which weighed and extracted with acetic/nitric reagent. The samples were then hydrolyzed with 67% sulfuric acid and the released glucose was quantified with anthrone reagent (Fisher).

### Histological analysis

For calcofluor white staining, the second leaf blades were fixed in Dietrich’s Formalin Acetic Acid (FAA) overnight at 4 °C. Fixed samples were processed with the aid of a Pelco BioWave Pro laboratory microwave (Ted Pella). Samples were dehydrated in a graded ethanol series, from 75%, 85%, 95%, to 100%. Dehydrated samples were infiltrated in LRWhite Hard resin at 50% then at 100% and cured at 100 °C for 24 h. Semi-thick sections (500 nm) were stained with Calcofluor-white (Sigma) for one minute followed by mounting sections to slides with Depex mounting medium and viewing under UV using an Olympus BX 51 upright fluorescence microscope.

For lignin staining, hand-cut specimens prepared from leaf blades were incubated in 2% (w/v) phloroglucinol-HCl for 5 min and viewed using an Olympus BX 51 upright fluorescence microscope.

## Supplementary information

Supplementary Information.

## Data Availability

The datasets generated and analyzed during the current study are available from the corresponding author on reasonable request.
