# Supplementary material for: Rice immune sensor XA21 differentially enhances plant growth and survival under distinct levels of drought
Source: Sci Rep. 2020 Oct 9;10:16938. doi: 10.1038/s41598-020-73128-7 (PMC7547014; doi:10.1038/s41598-020-73128-7)
Supplement: Supplementary file 1 — Supplementary Information. [file 41598_2020_73128_MOESM1_ESM.pdf]

SUPPLEMENTARY MATERIAL

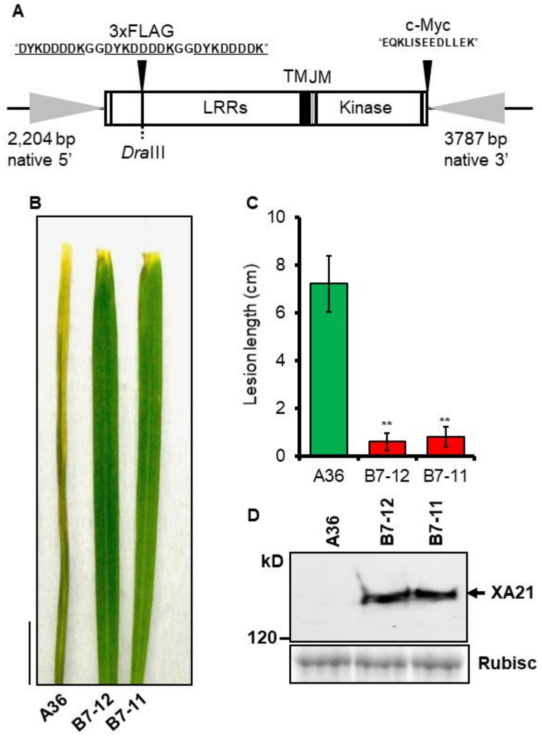

Supplementary Figure 1. A double-tagged XA21 confers resistance to *Xoo* PXO99^{A}. **(A)** Schematic representation of a double-tagged XA21. Domains of XA21 are as described previously[16]. LRRs, leucine-rich repeats; TM, transmembrane domain; JM, juxtamembrane domain. A triple FLAG tag was inserted into the *Dra* III site, whereas a c-Myc tag was fused in frame to the C-terminus of XA21. **(B)** Indicated transgenic lines showing lesion development 12 days after inoculation with *Xoo* PXO99^{A}. A36, empty-vector control. B7-12 and B7-11, independent lines transformed with the construct shown in A. Plants were inoculated at the 2-week-old stage. Scale bar = 1 cm. **(C)** Lesion length data of inoculated plants. Error bars are SD (n = 11). **, $P < 0.01$. **(D)** Protein blot analysis showing the levels of XA21 in the indicated transgenic plants. Identical protein samples were immuno-probed with anti-c-Myc (Top) or resolved by SDS-PAGE followed by Coomassie Brilliant Blue staining as a loading control (Bottom).

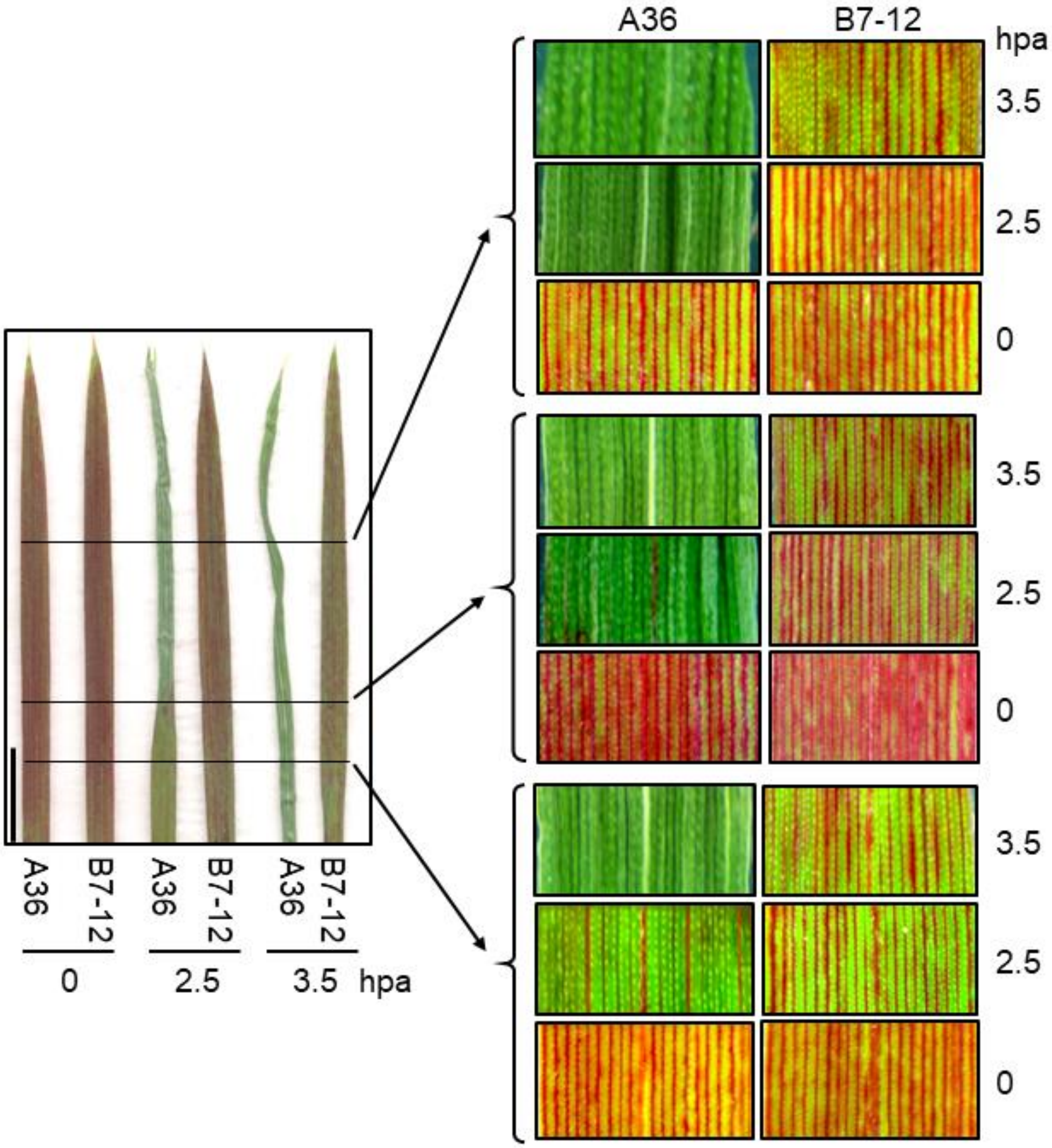

Supplementary Figure 2. Close-up images of Figure 4C. Pictures were taken in different positions along the leaves using a stereo microscope. Red color shows safranin-filled veins. Scale bar = 1 cm. hpa, hour post air-drying.

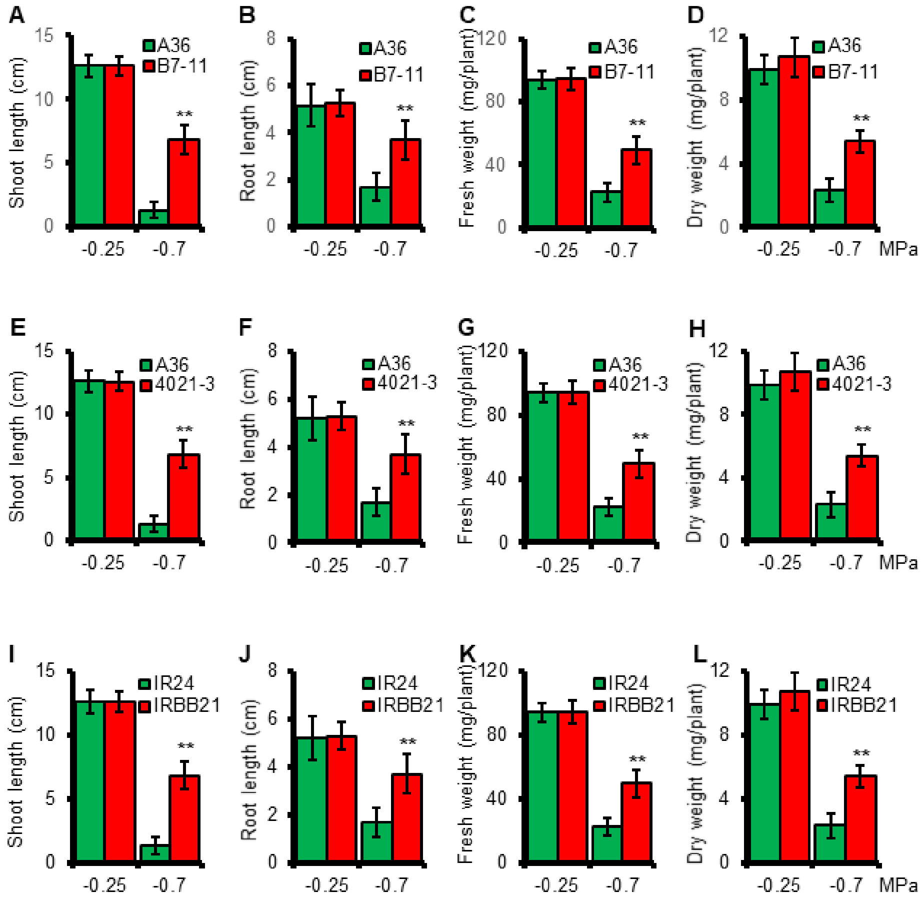

Supplementary Figure 3. XA21 is required for enhanced growth under low-ψ_w stress. Three-day-old rice seedlings of XA21 (B7-11, 4021-3 and IRBB21) and control (A36, IR24) lines (n = 10 per line) were transferred from 1/2 MS media (-0.25 MPa) to PEG-infused low-ψ_w agar plates (-0.7 MPa). Growth parameters were scored 5 days after transfer. **(A, B, E, F, I, J)** Shoot and root lengths of low-ψ_w treated seedlings. **(C, D, G, H, K, L)** Fresh and dry weights of low-ψ_w treated seedlings. Error bars are SD (n = 3). **, $P < 0.01$.

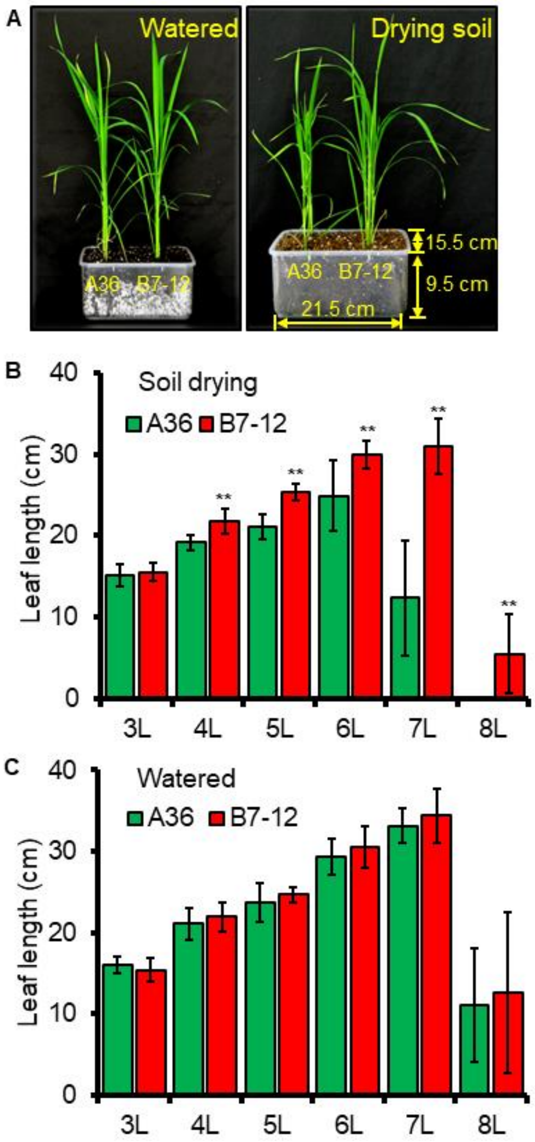

Supplementary Figure 4. XA21 is required for enhanced growth under moderate water-deficit stress. **(A)** Phenotypes of representative plants grown in well-watered soil or partially dry soil for one month. A total of six plants (three per line) were grown in each container. **(B-C)** Lengths of the 3rd (3L) to 8th (8L) leaf blades of well-watered and soid dryiny plants. Error bars in (**B**) and (**C**) are SD (n = 3). **, $P < 0.01$.

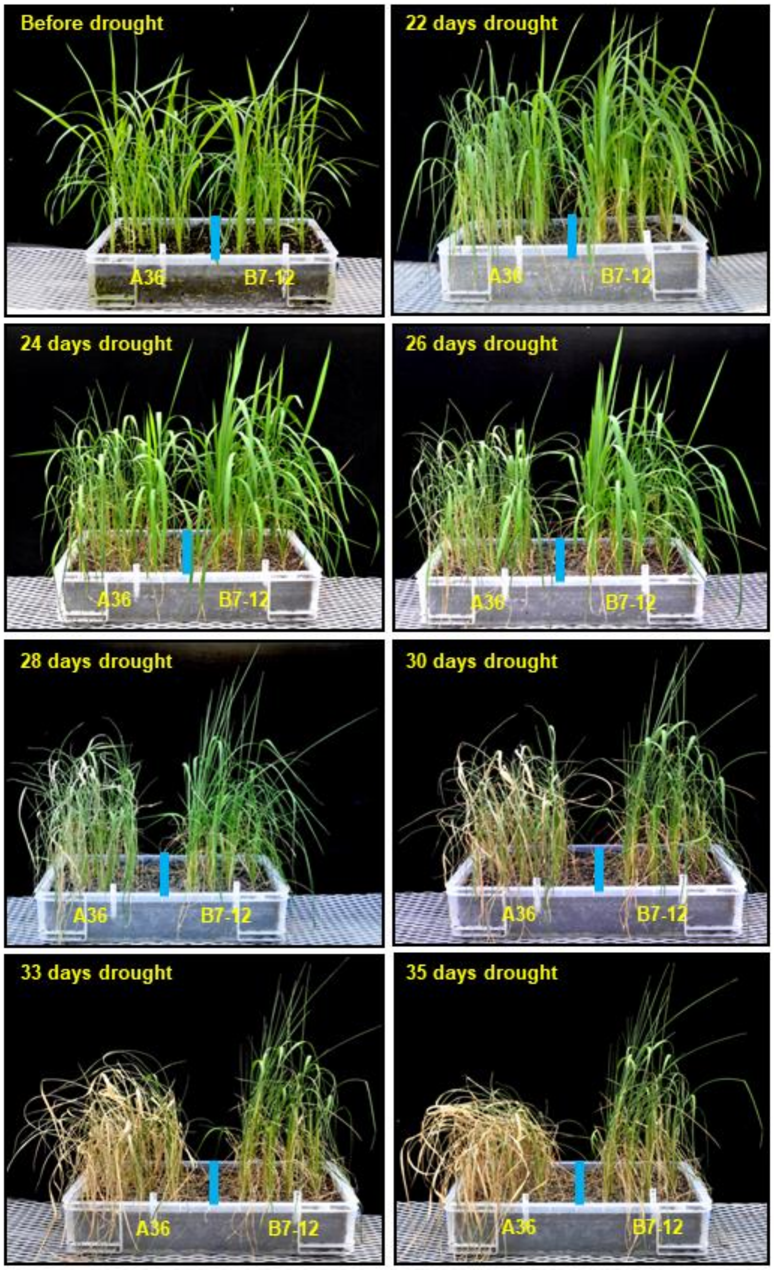

Supplementary Figure 5. Delayed leaf rolling and leaf death in the XA21 line B7-12 during drought stress (withheld water) treatments. The treated plants are the same as those in Figure 6A, except for showing phenotypes at more time points during stress.

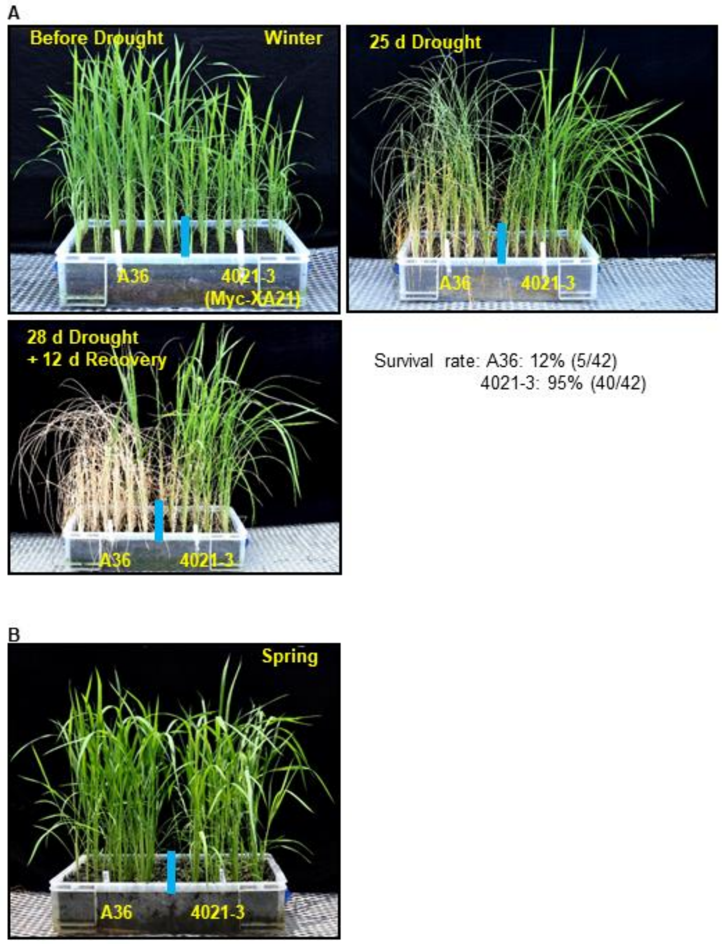

Supplementary Figure 6. The Myc-XA21 line 4021-3 displays enhanced drought survival under greenhouse conditions. **(A)** Phenotypes of 1-month-old XA21 plants (4021-3) and control (A36) (n = 42 each line) prior to, during and after drought stress treatments (withheld water) in winter. The survival rates for each line are indicated. **(B)** Phenotypes of 1-month-old 4021-3 and A36 plants (n = 16 each line) grown in the same greenhouse as in (**A**) during April and May. Of note, the retarded growth of 4021-3 in winter was not correlated with the density of plants grown in the container.

**Supplementary Table 1. Primer sequences for q-PCR and cloning**			
**Gene Name and ID**	**Forward primer (5'-3')**	**Reverse primer (5'-3')**	**Reference**
OsLEA1 (Os04g49980)	GTACATCTAGATTTGGGGTAGA	GTACGAACACAAGCTAACACGA	this study
OSLEA3 (*Os05g46480*)	CCAAGCAGAAGACCGCCGA	GTCATCCCCAGCGTGCTCA	this study
OsLEA33 (Os06g23350)	CGATGACGACGCTGAGTGAA	CAGGTGACATCACACGCTTGA	this study
OsNAC10 (*Os11g03300*)	TAACAGCACCACCACCACAA	GTCTTCAAGCTGTTCGACGG	this study
Os06g11170	GGAATGTGGACGGTGACACT	TCAAAATAGAGTCCAGTAGATTTGTCA	37
XA21-Tail	CTTTCCGAAGACGAGTATATCTAAC G	ACTAGTGGTACCCGTCTTATATCGCCT CA	this study
